# Predicting the subcellular location of prokaryotic proteins with DeepLocPro

**DOI:** 10.1093/bioinformatics/btae677

**Published:** 2024-11-14

**Authors:** Jaime Moreno, Henrik Nielsen, Ole Winther, Felix Teufel

**Affiliations:** Department of Biology, University of Copenhagen, 2200 Copenhagen, Denmark; AI & Digital Research, Novo Nordisk A/S, 2760 Måløv, Denmark; Department of Health Technology, Technical University of Denmark, 2800 Lyngby, Denmark; Department of Biology, University of Copenhagen, 2200 Copenhagen, Denmark; Department of Applied Mathematics and Computer Science, Technical University of Denmark, 2800 Lyngby, Denmark; Department of Biology, University of Copenhagen, 2200 Copenhagen, Denmark; AI & Digital Research, Novo Nordisk A/S, 2760 Måløv, Denmark

## Abstract

**Motivation:**

Protein subcellular location prediction is a widely explored task in bioinformatics because of its importance in proteomics research. We propose DeepLocPro, an extension to the popular method DeepLoc, tailored specifically to archaeal and bacterial organisms.

**Results:**

DeepLocPro is a multiclass subcellular location prediction tool for prokaryotic proteins, trained on experimentally verified data curated from UniProt and PSORTdb. DeepLocPro compares favorably to the PSORTb 3.0 ensemble method, surpassing its performance across multiple metrics in our benchmark experiment.

**Availability and implementation:**

The DeepLocPro prediction tool is available online at https://ku.biolib.com/deeplocpro and https://services.healthtech.dtu.dk/services/DeepLocPro-1.0/.

## 1 Introduction

Protein subcellular location is an important aspect of protein function within cells. It is directly related to the biochemical function of proteins, and it can provide valuable insights into the roles that they play in cellular processes, as well as aid in the design of biotechnological applications (McKay and Baldwin 1990, Schiraldi *et al.* 2002).

Numerous machine learning (ML) methods have been developed for predicting the subcellular location of eukaryotic proteins (Blum *et al.* 2009, Briesemeister *et al.* 2009, Almagro Armenteros *et al.* 2017). However, the availability of protein subcellular location predictors which address the distinct prediction problem of locations that are present in prokaryotic cells is more limited (Yu *et al.* 2004, 2010, Goldberg *et al.* 2012). While some works have been proposed in recent years (Wan *et al.* 2017, Grasso et al. 2021), implementations are often defunct and no longer available to the public. For an overview of the field, we refer to a recent review by one of us (Nielsen, 2024). In recent years, deep learning (DL) algorithms have become the method of choice for location prediction methods. However, these methods are either eukaryote specific (Stärk *et al.* 2021, Thumuluri *et al.* 2022) or not publicly available (Singh *et al.* 2022, Arora *et al.* 2024).

To bridge this gap, we introduce DeepLocPro, a DL method designed specifically for predicting the subcellular location of prokaryotic proteins. DeepLocPro utilizes protein language models (pLMs), which are neural networks that have been trained on large amounts of protein sequence data to learn ‘the language of proteins’. By leveraging pLMs, DeepLocPro is able to capture complex patterns in proteins, such as biochemical characteristics or evolutionary information encoded in the sequences that are indicative of the final subcellular location.

DeepLocPro has been trained to work with prokaryotic proteins from a wide range of organisms covering Archaea, Gram-positive bacteria, and Gram-negative bacteria. This adaptability renders it a versatile and applicable tool across multiple fields, including microbiology and biotechnology (Schiraldi *et al.* 2002, Drider and Rebuffat 2011).

## 2 Materials and methods

### 2.1 Data

**Figure 1.**
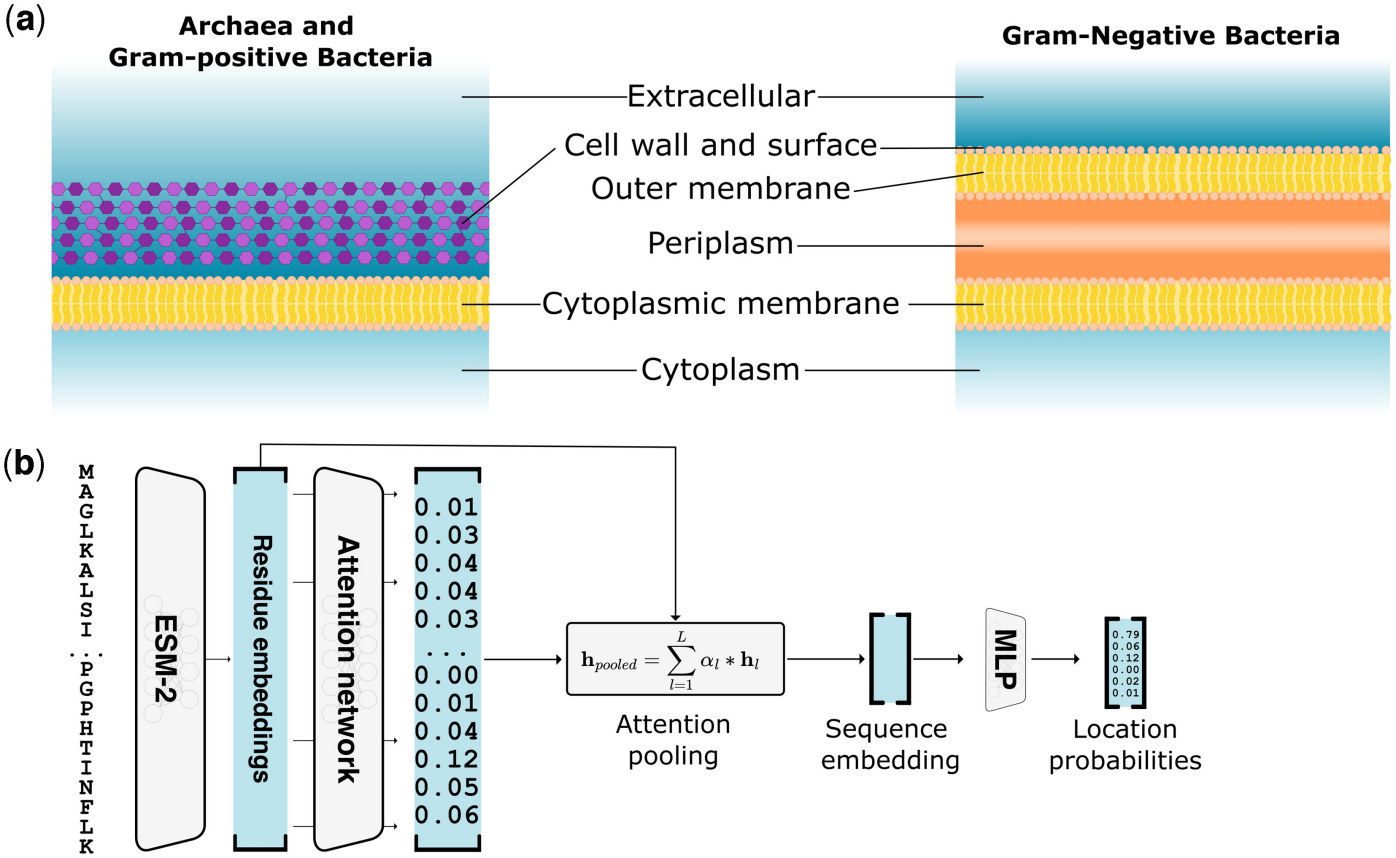
Modeling prokaryotic subcellular location. (a) Prokaryotic subcellular locations for archaea, Gram-positive and Gram-negative bacteria as modeled by DeepLocPro. (b) DeepLocPro model architecture. Sequences are embedded by the ESM-2 pLM, followed by an attention network that predicts a weight for each residue, which is then used to compute a weighted sum of all residue embeddings. The resulting sequence embedding serves as input to the classification layer that predicts location probabilities.

We curated experimentally verified subcellular location data from two sources: PSORTdb 4.0 (Lau *et al.* 2021) and UniProt release 2023_03 (The UniProt Consortium 2023). For the PSORTdb dataset, we exclusively considered experimentally verified entries and excluded proteins with unknown IDs or those that could not be mapped to Uniprot. Our focus was on six main subcellular locations for prokaryotic proteins: cell wall and surface, extracellular space, cytoplasm, cytoplasmic membrane, outer membrane, and periplasm. We retrieved the sequences of PSORTdb entries from UniProt, resulting in a dataset of 10 241 proteins. From UniProt, we curated proteins with experimentally verified subcellular locations belonging to taxonomies Archaea (ID:2157) and Bacteria (ID:2), yielding 2982 proteins. UniProt locations were mapped to the six main locations (Supplementary Table S1). To classify bacterial data into the Gram-positive and Gram-negative categories, we followed the methodology of SignalP 6.0 (Teufel *et al.* 2022), which defined Gram-positive as *monoderm* phyla and Gram-negative as *diderm* (Figure 1a). Both datasets were merged, removing duplicated entries and proteins with a sequence length of fewer than 40 amino acids.

Although multilabel prediction is desirable for this task (Thumuluri *et al.* 2022), there is not currently enough data to train the model as multilabel, as sequences with more than one location only made up a small fraction (∼3%) of the data. We therefore chose to treat the problem as a single location multi-class problem and remove the multilabel sequences from the dataset. A total of 11 970 protein sequences were obtained (Supplementary Table S2).

To measure model performance reliably on held out data, we homology partitioned our dataset into five-folds using GraphPart (Teufel *et al.* 2023). Sequences in a fold have a maximum 30% Needleman−Wunsch sequence identity to any sequence in other folds. Folds were balanced for organism groups and subcellular locations using GraphPart. As a consequence, 64 proteins were removed from the dataset to achieve separation of the five-folds (Table 1; Supplementary Table S3).

**Table 1.** The DeepLocPro training dataset.^a^

Group	Archaea	Negative	Positive	Total
Cell wall and surface	4	21	62	87
Extracellular	21	581	475	1077
Cytoplasmic	124	4700	2061	6885
Cytoplasmic Membrane	134	1793	608	2535
Outer Membrane	−	756	−	756
Periplasmic	−	566	−	566
Total	283	8417	3206	11 906

a Number of sequences per location returned by GraphPart categorized by organism group.

**Table 2.** Metrics of DeepLocPro.^a^

Metric	Count	Overall	Archaea	Gram pos	Gram neg
		(*n* = 11 906)	(*n* = 283)	(*n* = 3206)	(*n* = 8417)
Accuracy	−	0.92 ± 0.01	0.88 ± 0.05	0.93 ± 0.01	0.91 ± 0.01
Macro F1	−	0.80 ± 0.02	0.64 ± 0.19	0.82 ± 0.03	0.84 ± 0.04
Multiclass MCC	−	0.86 ± 0.01	0.79 ± 0.09	0.88 ± 0.02	0.85 ± 0.02
MCC per location					
Cytoplasmic	6885	0.91 ± 0.01	0.82 ± 0.07	0.92 ± 0.02	0.91 ± 0.02
Cytoplasmic membrane	2535	0.91 ± 0.01	0.82 ± 0.07	0.92 ± 0.02	0.91 ± 0.02
Extracellular	1077	0.81 ± 0.03	0.55 ± 0.39	0.84 ± 0.03	0.79 ± 0.07
Periplasmic	566	0.78 ± 0.05	−	−	0.78 ± 0.05
Outer membrane	756	0.75 ± 0.02	−	−	0.75 ± 0.02
Cell wall and surface	87	0.59 ± 0.08	0.00 ± 0.00	0.56 ± 0.11	0.82 ± 0.12

a Performance was calculated using nested cross-validation.

### 2.2 Model

The DeepLocPro model is based on the DeepLoc 2.0 architecture (Thumuluri *et al.* 2022). The DeepLoc 2.0 model performs attention pooling of a protein sequence embedding, followed by a multilayer perceptron (MLP) for predicting class probabilities. As the embedding model, we used the publicly available pLM ESM2 with 650M parameters (Lin *et al.* 2023). We simplified the DeepLoc 2.0 architecture by omitting the sorting signal prediction module and training without regularization of attention weights. As DeepLocPro is a multiclass model, the sigmoidal activation function of the output layer was replaced with a Softmax (Figure 1b).

The neural network itself is agnostic to the organism group of origin, relying on the pLM’s ability to correctly infer the origin organism group from the sequence alone (Alley *et al.* 2019, Elnaggar *et al.* 2022, Teufel *et al.* 2022). This circumvents the requirement to separately predict the organism group if it is not known, as done for metagenomic reads in PSORTm (Peabody *et al.* 2020). Optionally, to avoid spurious predictions, DeepLocPro can be provided with the organism group information. If this information is provided, biologically implausible predictions (outer membrane and periplasmic proteins in archaea and Gram-positive organisms) are remapped to extracellular, as those locations lie beyond the inner membrane. When applied to metagenomic data, DeepLocPro requires the N-terminus of the protein to be complete, and should not be run on raw reads.

For training, we used Adam as the optimizer and Cross Entropy loss. We also implemented a dynamic learning rate reduction strategy, decreasing the learning rate by a factor of 0.1 when the model stopped improving for five epochs. Models were trained for a total of 60 epochs. The model was implemented in PyTorch.

### 2.3 Evaluation

The model was evaluated using five-fold nested cross-validation. Having five dataset folds, each outer loop iteration involves selecting one-fold as the test set, and combining the remaining four-folds in different combinations for training and validation. These four different combinations constitute the inner loop. In total, 20 models are trained.

We optimized the model's hyperparameters by running the model with different combinations of learning rates (0.1, 0.01, 0.001), batch sizes (8, 16, 32, 64), and dropout rates (0.1, 0.2, 0.4). For each fold, we selected the best performing hyperparameters by calculating the F1 macro average metric for the four possible inner iterations within each fold (Supplementary Table S4).

After hyperparameter optimization, the final performance of the model was reported by averaging the class probabilities of each four inner loop models on their test fold. We thereby obtained a held-out test prediction for each sample in our dataset. We computed performance metrics on each fold, reporting the average metric and standard deviation over five-folds. We assessed the performance using three metrics: Accuracy, Macro F1, and Matthews Correlation Coefficient (MCC, multiclass and single class) (Gorodkin 2004).

### 2.4 Benchmark

For comparison, we report performance of PSORTb 3.0 (Yu *et al.* 2010), which we consider a reference method for prokaryotic subcellular location prediction due to its popularity and strong performance compared to other methods (Magnus *et al.* 2012). PSORTb 3.0 is an ensemble model that combines the output of multiple prediction methods. Beyond a collection of ML predictors, it also features homology-based components such as a BLAST search and sequence motifs.

While homology is a powerful mechanism for the assignment of locations to new sequences, it presents a challenge for comparing the performance of ML methods. If a test sequence is similar to the labeled data underlying the homology modules, it can be trivially classified as correct by the method. However, this performance will not be indicative of the true test performance that can be expected on new data that are dissimilar to the known training data. To mitigate such effects, we (i) disabled the SCLBLAST module of PSORTb 3.0 (Supplementary Note 1) and (ii) created a benchmark test subset of sequences that were not available at the creation of PSORTb 3.0, using a temporal cutoff, including sequences from 2010 onward, since it was when the model was released.

While these steps somewhat reduce performance overestimation, we note that the reported performance can still be considered overestimated, given that—contrary to the DeepLocPro evaluation—it is not possible to guarantee that the data used for testing has a maximum 30% sequence identity to the data used to train the available PSORTb 3.0 ensemble predictor. Unlike DeepLocPro, which always returns a prediction for any given protein, PSORTb 3.0 can return “Unknown” as a location prediction. For computing performance metrics, unknown predictions count as false negatives.

## 3 Results

### 3.1 DeepLocPro performance

In nested cross-validation, DeepLocPro achieved an overall accuracy of 0.92 and an overall macro F1 score of 0.80 (Table 2). The performance of DeepLocPro varied across different subcellular locations. We observed the highest performance on cytoplasmic and cytoplasmic membrane localized proteins, with an MCC of 0.91 and 0.88, respectively. We find that predicting locations with a small number of training samples, such as the cell wall and surface, remains challenging with an MCC of 0.59. For cell wall and surface, we attribute this to the fact that the class is heterogeneous and largely exhibits shared biological features with the extracellular class, which makes it hard to learn the distinguishing characteristics of the two groups (Supplementary Figure S1).

Stratification of performance by organism groups revealed that prediction of location in archaea is more challenging than in bacteria. The prediction of cell wall sequences shows an MCC of −0.01, indicating that DeepLocPro has random performance. We attribute this to the fact that there are only four sequences available for this category, and the archaeal cell wall being distinct from bacterial cell walls (Albers and Meyer 2011), so that transfer learning from the more numerous bacterial sequences does not take place.

Additionally, we computed confusion matrices for all organism groups (Supplementary Figure S1). We observe that distinguishing between cell wall and surface and extracellular proteins still remains challenging. This is likely due to the fact that both locations make use of the secretory pathway, while the sequence features that localize a protein to the cell wall or surface after secretion are more elusive and varied.

### 3.2 Benchmarking results

On the post-2010 benchmark set, DeepLocPro outperformed PSORTb 3.0 in all three groups in terms of accuracy, macro F1 score, and multiclass MCC (Table 3). Per-location performance metrics reveal that DeepLocPro outperformed PSORTb in all subcellular locations in the Gram-positive and -negative groups, which contains the largest number of sequences. In archaea, PSORTb surpassed DeepLocPro’s performance for extracellular proteins.

**Table 3.** Performance of DeepLocPro and PSORTb 3.0 on the benchmark set.

		Archaea (*n* = 131)		Gram positive (*n* = 366)		Gram negative (*n* = 634)	
Metric	Count	DeepLocPro	PSORTb	DeepLocPro	PSORTb	DeepLocPro	PSORTb
Accuracy	−	**0.89**	0.70	**0.79**	0.50	**0.74**	0.34
Macro F1	−	**0.56**	0.51	**0.66**	0.48	**0.75**	0.35
Multiclass MCC	−	**0.79**	0.49	**0.70**	0.41	**0.69**	0.30
MCC per location							
Cell wall and surface	35	−	−	**0.28**	–0.02	**0.80**	0.00
Extracellular	288	0.42	**0.57**	**0.70**	0.44	**0.76**	0.28
Cytoplasmic	250	**0.81**	0.54	**0.67**	0.55	**0.68**	0.36
Cytoplasmic membrane	325	**0.85**	0.65	**0.78**	0.51	**0.69**	0.59
Outer membrane	160	−	−	−	−	**0.60**	0.28
Periplasmic	73	−	−	−	−	**0.72**	0.34

Best value highlighted in bold.

## 4 Discussion

The accurate prediction of subcellular location is critical for understanding the function and interactions of proteins within a cell. In this study, we developed DeepLocPro, an ML method for predicting the subcellular location of prokaryotic proteins using protein language models.

Combining experimentally verified data from two sources, we established a comprehensive dataset of over 11 900 sequences. However, for some locations such as the archaeal cell wall and surface, the number of available experimentally verified proteins is still severely limiting for training ML algorithms. While pLMs can help mitigate limited data to some extent, we find that model performance still correlates with the availability of training sequences. This indicates that future work might benefit from targeted data curation for the locations that are underrepresented in the databases. Even though comparing the performance of DeepLocPro directly to PSORTb 3.0 is challenging, as it cannot be evaluated at a sequence identity threshold of 30%, our findings indicate that DeepLocPro’s cross-validated predictions outperform PSORTb 3.0 on all but one metric, showing a strong improvement in the performance especially for bacterial proteins.

## 5 Conclusion

We introduce DeepLocPro, a multiclass subcellular location prediction tool for prokaryotic proteins. The model is based on pretrained protein language models that capture biochemical and evolutionary information, resulting in improved performance compared to current methods. The DeepLocPro web server is accessible online at https://ku.biolib.com/deeplocpro and https://services.healthtech.dtu.dk/services/DeepLocPro-1.0/.

## Supplementary Material

btae677_Supplementary_Data

## Data Availability

The training and benchmark datasets are available at https://services.healthtech.dtu.dk/services/DeepLocPro-1.0/. The codebase for training DeepLocPro is available at https://github.com/Jaimomar99/DeepLocPro_model.

## References

[btae677-B1] Albers S-V , Meyer BH. The archaeal cell envelope. Nat Rev Microbiol 2011;9:414–26. 10.1038/nrmicro2576 21572458

[btae677-B2] Alley EC , Khimulya G, Biswas S et al Unified rational protein engineering with sequence-based deep representation learning. Nat Methods 2019;16:1315–22. 10.1038/s41592-019-0598-1 31636460 PMC7067682

[btae677-B3] Almagro Armenteros JJ , Sønderby CK, Sønderby SK et al DeepLoc: prediction of protein subcellular localization using deep learning. Bioinformatics 2017;33:3387–95. 10.1093/bioinformatics/btx431 29036616

[btae677-B4] Arora I , Kummer A, Zhou H et al mtx-COBRA: subcellular localization prediction for bacterial proteins. Comput Biol Med 2024;171:108114. 10.1016/j.compbiomed.2024.108114 38401450

[btae677-B5] Blum T , Briesemeister S, Kohlbacher O. MultiLoc2: integrating phylogeny and gene ontology terms improves subcellular protein localization prediction. BMC Bioinformatics 2009;10:274. 10.1186/1471-2105-10-274 19723330 PMC2745392

[btae677-B6] Briesemeister S , Blum T, Brady S et al SherLoc2: a high-accuracy hybrid method for predicting subcellular localization of proteins. J Proteome Res 2009;8:5363–6. 10.1021/pr900665y 19764776

[btae677-B7] Drider D , Rebuffat S. 2011. Prokaryotic Antimicrobial Peptides: From Genes to Applications. New York: Springer Science & Business Media.

[btae677-B8] Elnaggar A , Heinzinger M, Dallago C et al ProtTrans: toward understanding the language of life through self-supervised learning. IEEE Trans Pattern Anal Mach Intell 2022;44:7112–27. 10.1109/TPAMI.2021.3095381 34232869

[btae677-B9] Goldberg T , Hamp T, Rost B. LocTree2 predicts localization for all domains of life. Bioinformatics 2012;28:i458–65. 10.1093/bioinformatics/bts390 22962467 PMC3436817

[btae677-B10] Gorodkin J. Comparing two K-category assignments by a K-category correlation coefficient. Comput Biol Chem 2004;28:367–74. 10.1016/j.compbiolchem.2004.09.006 . 15556477

[btae677-B11] Grasso S , van Rij T, van Dijl JM. GP^4^: an integrated Gram-positive protein prediction pipeline for subcellular localization mimicking bacterial sorting. Brief Bioinform 2021;22:bbaa302. 10.1093/bib/bbaa302 33227814 PMC8294519

[btae677-B12] Lau WYV , Hoad GR, Jin V et al PSORTdb 4.0: expanded and redesigned bacterial and archaeal protein subcellular localization database incorporating new secondary localizations. Nucleic Acids Res 2021;49:D803–8. 10.1093/nar/gkaa1095 33313828 PMC7778896

[btae677-B13] Lin Z , Akin H, Rao R et al Evolutionary-scale prediction of atomic-level protein structure with a language model. Science 2023;379:1123–30. 10.1126/science.ade2574 36927031

[btae677-B14] Magnus M , Pawlowski M, Bujnicki JM. MetaLocGramN: a meta-predictor of protein subcellular localization for gram-negative bacteria. Biochim Biophys Acta 2012;1824:1425–33. 10.1016/j.bbapap.2012.05.018 22705560

[btae677-B15] McKay LL , Baldwin KA. Applications for biotechnology: present and future improvements in lactic acid bacteria. FEMS Microbiol Rev 1990;7:3–14. 10.1111/j.1574-6968.1990.tb04876.x 2271224

[btae677-B16] Nielsen H. Protein sorting prediction. In: Journet L., Cascales E. (eds), Bacterial Secretion Systems: Methods and Protocols. New York, US: Springer, 2024, 27–63. 10.1007/978-1-0716-3445-5_2

[btae677-B0970994] Peabody MA, , Lau WYV, , Hoad GR et al PSORTm: A bacterial and archaeal protein subcellular localization prediction tool for metagenomics data. Bioinformatics 2020;36:3043–8. 10.1093/bioinformatics/btaa136 32108861 PMC7214030

[btae677-B17] Schiraldi C , Giuliano M, De Rosa M. Perspectives on biotechnological applications of archaea. Archaea 2002;1:75–86. 10.1155/2002/436561 15803645 PMC2685559

[btae677-B18] Singh G , Tyagi R, Singh A et al Protein language model for prediction of subcellular localization of protein sequences from gram-negative bacteria (ProtLM.SCL). bioRxiv, 10.1101/2022.12.16.520742, 2022, preprint: not peer reviewed.

[btae677-B19] Stärk H , Dallago C, Heinzinger M et al Light attention predicts protein location from the language of life. Bioinform Adv 2021;1:vbab035. 10.1093/bioadv/vbab035 36700108 PMC9710637

[btae677-B20] Teufel F , Almagro Armenteros JJ, Johansen AR et al SignalP 6.0 predicts all five types of signal peptides using protein language models. Nat Biotechnol 2022;40:1023–5. 10.1038/s41587-021-01156-3 34980915 PMC9287161

[btae677-B21] Teufel F , Gíslason MH, Almagro Armenteros JJ et al GraphPart: homology partitioning for biological sequence analysis. NAR Genom Bioinform 2023;5:lqad088. 10.1093/nargab/lqad088 37850036 PMC10578201

[btae677-B22] The UniProt Consortium. UniProt: the universal protein knowledgebase in 2023. Nucl Acids Res 2023;51:D523–31. 10.1093/nar/gkac1052 36408920 PMC9825514

[btae677-B23] Thumuluri V , Almagro Armenteros JJ, Johansen AR et al DeepLoc 2.0: multi-label subcellular localization prediction using protein language models. Nucleic Acids Res 2022;50:W228–34. 10.1093/nar/gkac278 35489069 PMC9252801

[btae677-B24] Wan S , Mak M, Kung S. Gram-LocEN: interpretable prediction of subcellular multi-localization of Gram-positive and Gram-negative bacterial proteins. Chemom Intell Lab Syst 2017;162:1–9. 10.1016/j.chemolab.2016.12.014

[btae677-B25] Yu C-S , Lin C-J, Hwang J-K. Predicting subcellular localization of proteins for Gram-negative bacteria by support vector machines based on n-peptide compositions. Protein Sci A Publ Protein Soc 2004;13:1402–6. 10.1110/ps.03479604 PMC2286765 15096640

[btae677-B26] Yu NY , Wagner JR, Laird MR et al PSORTb 3.0: improved protein subcellular localization prediction with refined localization subcategories and predictive capabilities for all prokaryotes. Bioinformatics 2010;26:1608–15. 10.1093/bioinformatics/btq249 20472543 PMC2887053

