# Supplementary material for: Predicting the subcellular location of prokaryotic proteins with DeepLocPro
Source: Bioinformatics. 2024 Nov 14;40(12):btae677. doi: 10.1093/bioinformatics/btae677 (PMC11645106; doi:10.1093/bioinformatics/btae677)
Supplement: btae677_Supplementary_Data [file btae677_supplementary_data.pdf]

Predicting the subcellular location of prokaryotic proteins with DeepLocPro

Jaime Moreno[12], Henrik Nielsen[3], Ole Winther[14], Felix Teufel[12]
1 Department of Biology, University of Copenhagen
2 AI and Digital Research, Digital Science & Innovation, Novo Nordisk A/S
3 Department of Health Technology, Technical University of Denmark
4 Department of Applied Mathematics and Computer Science, Technical University of Denmark

Supplementary Data

UniProt location	Model location
Cell surface	Cell wall and surface
Spore cortex	Cell wall and surface
Spore wall	Cell wall and surface
Secreted	Extracellular
Host nucleus	Extracellular
Host membrane	Extracellular
Host endoplasmic reticulum	Extracellular
Host cytoplasm, host perinuclear region	Extracellular
Target cell, target cell cytoplasm	Extracellular
Cytoplasm	Cytoplasmic

UniProt location	Model location
Spore core	Cytoplasmic
Cellular thylakoid lumen	Cytoplasmic
Periplasm	Periplasmic
Forespore intermembrane space	Periplasmic
Forespore inner membrane	Cytoplasmic membrane
Cell inner membrane	Cytoplasmic membrane
Cellular thylakoid membrane	Cytoplasmic membrane
Cell membrane	Cytoplasmic membrane
Cell outer membrane	Outer membrane
Forespore outer membrane	Outer membrane

Table S1. **UniProt subcellular location mapping.** The original subcellular locations from Uniprot were mapped to the six location classes modeled by DeepLocPro.

Organis m Group	Cell wall and surface	Extracellular	Cytoplasmic	Cytoplasmic membrane	Outer membrane	Periplasmic	Total
**Archaea**	4	21	124	138	0	0	287
**Negative**	21	588	4717	1816	758	568	8468
**Positive**	62	476	2064	613	0	0	3215
**Total**	87	1085	6905	2567	758	568	11970

Table S2. **The DeepLocPro dataset before GraphPart partitioning into five folds at 30% maximum identity.**

Fold	Cell wall and surface	Extracellular	Cytoplasmic	Cytoplasmic membrane	Outer membrane	Periplasmic	Total
**0**	21	195	1540	525	163	110	2554
**1**	16	257	1638	522	136	115	2684
**2**	22	193	1167	487	134	95	2098
**3**	16	224	1568	476	159	136	2579
**4**	12	208	972	525	164	110	1991
**total**	87	1077	6885	2535	756	566	11906

Table S3. **Compositions of the five folds of the DeepLocPro dataset.**

Test fold	Learning rate	Batch size	Drop-out
**0**	0.001	16	0.4
**1**	0.001	32	0.2
**2**	0.001	16	0.4
**3**	0.001	32	0.2
**4**	0.001	16	0.4

Table S4. **Final hyperparameters found for each nested cross-validation outer loop.**

Metric	Overall	Archaea	Gram pos.	Gram neg.
Accuracy	0.92 ± 0.01	0.88 ± 0.05	0.93 ± 0.01	0.91 ± 0.01
Macro F1	0.80 ± 0.02	0.60 ± 0.21	0.57 ± 0.05	0.84 ± 0.04
Multiclass MCC	0.86 ± 0.01	0.79 ± 0.09	0.88 ± 0.02	0.85 ± 0.02
Cytoplasmic	0.91 ± 0.01	0.82 ± 0.07	0.92 ± 0.02	0.91 ± 0.02
Cytoplasmic Membrane	0.88 ± 0.02	0.86 ± 0.06	0.89 ± 0.02	0.88 ± 0.03
Extracellular	0.82 ± 0.03	0.58 ± 0.40	0.85 ± 0.03	0.79 ± 0.07
Periplasmic	0.78 ± 0.05	0.00 ± 0.00	0.00 ± 0.00	0.78 ± 0.05
Outer Membrane	0.75 ± 0.02	0.00 ± 0.00	0.00 ± 0.00	0.75 ± 0.02
Cell wall and surface	0.59 ± 0.08	0.00 ± 0.00	0.56 ± 0.11	0.82 ± 0.12

Table S5. **Model performance without using organism group information.** No remapping to extracellular is performed for periplasmic and outer membrane predictions in Archaea and Gram pos.

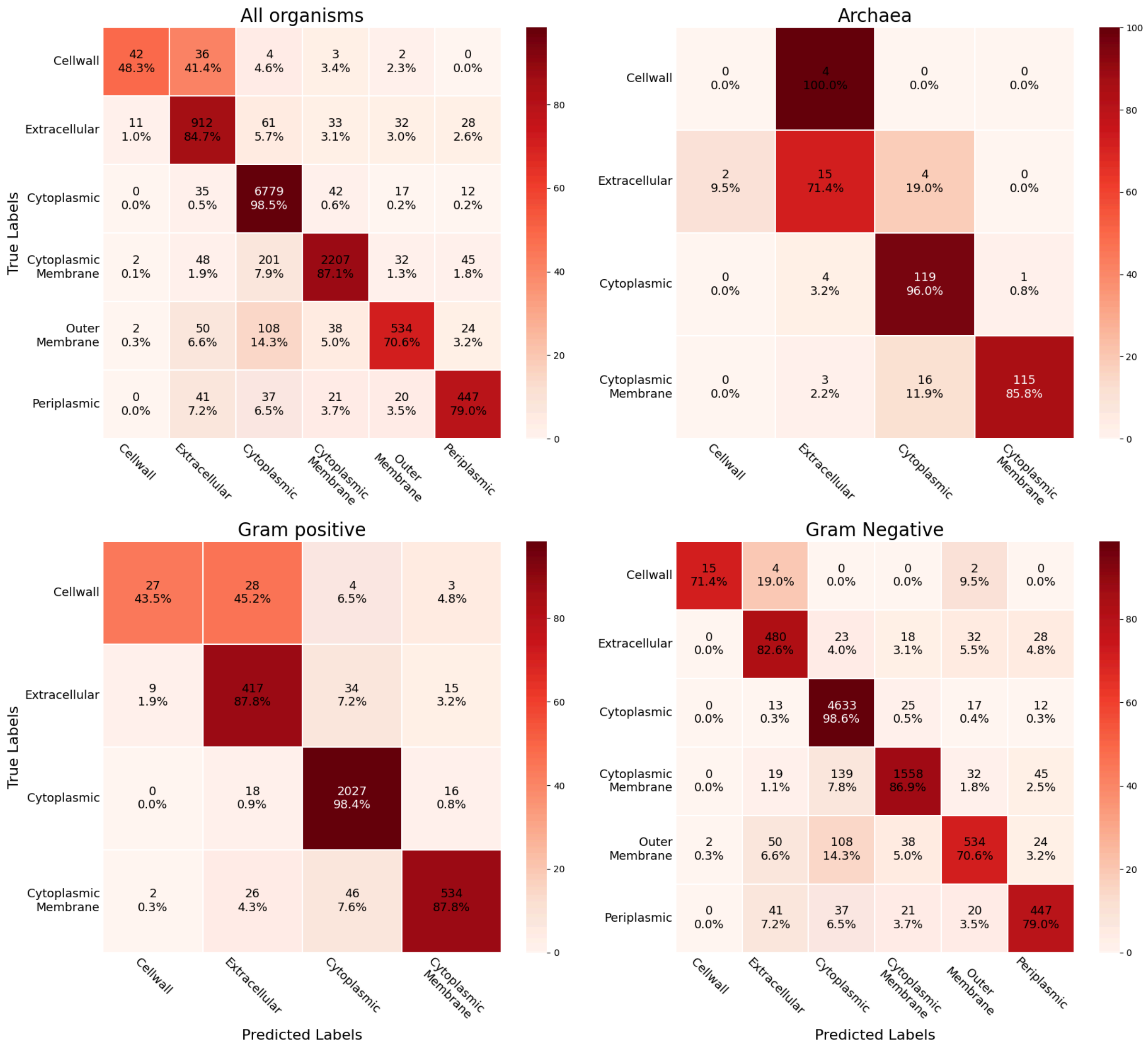

Figure S1. **Confusion matrices of DeepLocPro for each organism group**.

Supplementary Note 1. Disabling BLAST in PSORTb 3.0

As PSORTb 3.0 does not feature an option to disable the SCLBLAST module, we modified the `brinkmanlab/psortb_commandline:1.0.2` docker image. To avoid interfering with the source code of the method, we disabled BLAST functionality by emptying the BLAST databases used by the method using the following steps.

1. Launch the docker container in interactive mode
   ```
   sudo docker run -it --entrypoint /bin/bash
   brinkmanlab/psortb_commandline:1.0.2
   ```
2. Navigate to `/usr/local/psortb/conf/analysis/sclblast` and delete the contents of the files `archaea/sclblast`, `gramneg/sclblast` and `grampos/sclblast`
3. Run `sh makedb.sh` to regenerate the databases from the sclblast files
4. Exit the docker container and use `docker commit` to save a modified image `psortb_commandline_noblast`
5. Modify the psortb perl script to execute the new image by replacing lines 11 and 61 with
   ```
   our $docker_image = "psortb_commandline_noblast";
   my $cmd = "sudo docker run --rm -v
   $nondocker_results_dir:$docker_results_dir -e
   MOUNT='$nondocker_results_dir' --entrypoint
   /usr/local/psortb/bin/psort  -ti $docker_image
   /usr/local/psortb/bin/psort $extra_args -i $docker_seqfile";
   ```
